# Effect of Rho-Kinase and Autophagy on Remote Ischemic Conditioning-Induced Cardioprotection in Rat Myocardial Ischemia/Reperfusion Injury Model

**DOI:** 10.1155/2022/6806427

**Published:** 2022-01-06

**Authors:** Jie Gao, Feng Min, Shasha Wang, Heng Lv, Huan Liang, Ben Cai, Xianjie Jia, Qin Gao, Ying Yu

**Affiliations:** ^1^Department of Physiology, School of Basic Medicine, Bengbu Medical College, Bengbu 233000, China; ^2^Key Laboratory of Cardiovascular and Cerebrovascular Diseases, Bengbu Medical College, Bengbu 233000, China; ^3^Xishan District Center for Disease Control and Prevention, Wuxi 214000, China; ^4^Department of Epidemiology and Statistics, School of Public Health, Bengbu Medical College, Bengbu 233000, China

## Abstract

**Objective:**

Remote ischemic conditioning (RIC) is a cardioprotective method in ischemia/reperfusion (I/R) injury. This study investigated the mechanism of Rho-kinase-mediated autophagy in RIC.

**Methods:**

Sixty male Sprague–Dawley rats were randomly divided into six groups: sham, I/R, RIC, I/R+fasudil, RIC+wortmannin, and RIC+fasudil+wortmannin. Throughout the experiment, mean arterial pressure and heart rate were continuously monitored. Histopathology and ultrastructure and myocardial enzymes' expression were evaluated to determine the degree of cardiac injury. The protein expression of the Rho-kinase substrates myosin light chain (MLC) and myosin phosphatase target subunit 1 (MYPT1), autophagy-related protein light chain 3-II (LC3-II) and Beclin 1, and protein kinase B (AKT) was measured in the myocardial tissue.

**Results:**

Compared with the sham group, the mean arterial pressure and heart rate were decreased, myocardial enzyme levels were increased, and myocardial damage was aggravated in the I/R group; however, RIC improved these alterations. The expression of phosphorylated MLC and MYPT1 was lower, while LC3-II, Beclin 1, and phospho-AKT expression levels were higher in the RIC group compared with the I/R group. Obviously, treatment of the I/R group rats with fasudil, a Rho-kinase inhibitor, significantly ameliorated the I/R effects, whereas treatment of the RIC group rats with wortmannin, a phosphatidylinositol-3 kinase (PI3K) inhibitor, inhibited the RIC protective effects. Moreover, the rats in the RIC+fasudil+wortmannin group showed similar changes to those in the RIC+wortmannin group.

**Conclusion:**

These results showed that RIC protected the myocardium from I/R injury by suppressing Rho-kinase and the underlying mechanism may be related to enhancing autophagy via the PI3K/AKT pathway.

## 1. Introduction

Myocardial ischemia/reperfusion injury (MIRI), which often occurs in the course of reperfusion treatment of ischemic heart disease, exacerbates cell death, aggravates myocardial damage, and threatens the life of the patient [1]. Remote ischemic conditioning (RIC) is a novel therapy for MIRI [2]. RIC is a combination of remote ischemic preconditioning (RIPreC) and remote ischemic postconditioning (RIPostC) through tissues or organs distant from the heart. Transient I/R treatment before and after ischemia is an effective mechanism to reduce I/R injury [2–4]. Moreover, RIPreC and RIPostC can reduce myocardial infarct size, stabilize morphology, and restore function during MIRI [3, 5–7]. Therefore, a better understanding of the mechanism will help develop protective approaches for patients with MIRI.

Rho-kinase mediates cell contraction, apoptosis, and inflammation and is a crucial factor in the pathogenesis of most myocardial diseases, such as heart failure and MIRI [8–10]. Rho-kinase mediates phosphorylated myosin light chain (p-MLC) and phosphorylated myosin phosphatase target subunit 1 (p-MYPT1) to enhance myocardial injury. Inhibition of Rho-kinase activity is considered to be a therapeutic target for myocardial protection [11, 12]. Several studies have shown that RIPreC and RIPostC inhibit the expression of myocardial p-MLC and p-MYPT1, oxidative stress, and inflammatory infiltration [11–13]. The phosphatidylinositol-3 kinase/protein kinase B (PI3K/AKT) pathway is involved in myocardial protection. This pathway affects cellular oxidative stress, inflammation, and the balance between autophagy and apoptosis [14–17]. Our previous study showed that RIPostC had cardioprotective and antiapoptotic roles through activation of the PI3K/AKT signaling pathway [18]. Moreover, we observed that RIPostC attenuated I/R injury by inhibiting Rho-kinase activity [3]. However, whether the cardioprotective effect of RIC is related to downregulation of Rho-kinase activity and the PI3K/AKT signaling pathway remains unclear.

Autophagy is the basis for maintaining the stability of myocardial structure and function. Autophagy degrades damaged cells and tissues and recovers macromolecular substances through I/R injury [19–21]. Autophagy maintains intracellular homeostasis and provides cell protection. Light chain 3- (LC3-) II and Beclin 1 are usually monitored to determine the level of intracellular autophagy. Interestingly, autophagy has been reported to play a “double-edged sword” role in the process of I/R. Autophagy exerts protective effects during ischemia; however, excessive autophagy may exacerbate cell death under reperfusion [22, 23]. In recent years, autophagy has been recognized as a crucial factor in the process of RIC cardioprotection [24–26]. RIC treatment increases LC3-II and Beclin 1 expression, alleviates cell apoptosis, and enhances myocardial function [22, 27, 28]. Unfortunately, little is known about the complex regulatory mechanisms in RIC. A number of studies have shown that the regulation of autophagy is inseparable from the PI3K/AKT pathway [25, 26, 29]. Nevertheless, the ambiguous role of autophagy in myocardial RIC remains to be determined. Additionally, whether autophagy mediated by the PI3K/AKT pathway is involved in the development of myocardial RIC is still unknown.

Therefore, the objectives of the present study were as follows: (1) to investigate the role of Rho-kinase in RIC; (2) to determine whether RIC exerts its cardiac protective effect on autophagy through activation of the PI3K/AKT signaling pathway; and (3) to clarify whether RIC inhibits Rho-kinase and activates PI3K/AKT pathway-mediated autophagy to protect the myocardium during MIRI.

## 2. Materials and Methods

### 2.1. Animals

Sixty male Sprague–Dawley rats (weight: 300 ± 50 g) were acquired from the Laboratory Animal Center of Bengbu Medical College. All rats had a normal diet and fresh water with a 12 h light/dark cycle. And the rats were raised in individual cages with constant humidity (50%) and temperature (22°C–26°C). All experiment protocols were approved by the Animal Use and Care Committee of Bengbu Medical College (Ethical number: 2017-075).

### 2.2. Chemicals and Reagents

Wortmannin was purchased from Sigma-Aldrich (St. Louis, Missouri, USA). Fasudil was purchased from Red Sun Pharmaceutical (Tianjin, China). Creatinine kinase (CK) and lactate dehydrogenase (LDH) assay kits were purchased from Jiancheng Institute of Biotechnology (Nanjing, China). The cardiac troponin I (cTnI) enzyme-linked immunosorbent assay (ELISA) kit was purchased from Calvin Biotechnology (Suzhou, China). Primary antibodies against Beclin 1, AKT, and p-AKT were purchased from Abcam (Cambridge, UK). p-MLC, MLC, p-MYPT1, MYPT1, and LC3-II antibodies were purchased from Cell Signaling Technology (Danvers, Massachusetts, USA). Glyceraldehyde-3-phosphate dehydrogenase (GAPDH) antibody was purchased from Absin Bioscience Inc. (Shanghai, China).

### 2.3. Experimental Design

**Figure 1:**
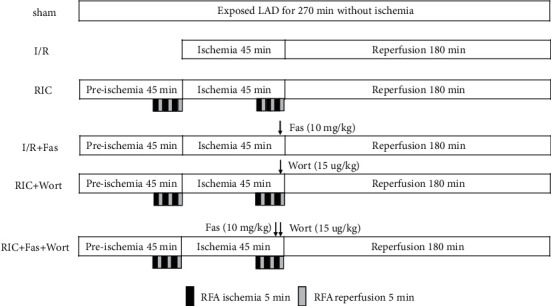
Experiment procedure. LAD: left anterior descending; RFA: right femoral artery; sham: LAD with no other intervention; I/R: ischemia/reperfusion; RIC: remote ischemic conditioning; Fas: fasudil; Wort: wortmannin.

Healthy rats were randomly assigned into six groups (*n* = 10 in each group) as follows: sham, I/R, RIC, I/R+fasudil (I/R+Fas), RIC+wortmannin (RIC+Wort), and RIC+fasudil+wortmannin (RIC+Fas+Wort). The I/R model was established in accordance with our previously reported method [3]. All rats were anesthetized by intraperitoneal injection of 4% chloral hydrate (10 ml/kg). The right common carotid artery was isolated, and the cannula was connected to continuously monitor the mean arterial pressure (MAP) and heart rate (HR) through the MedLab U/4C501Hbiological signal collecting system (Medease Science and Technology, Nanjing, China). After tracheal intubation, ventilation was applied via the ALC-V9 small animal ventilator (Alcott Biotech Corporation, Shanghai, China). The artificial respiration frequency was 70-80 breaths/minute, and the tidal volume was 20-30 mL/kg. Then, the left anterior descending (LAD) artery was ligated for 45 min, followed by 180 min of reperfusion. In the sham group, rats underwent thoracotomy for 270 min without LAD artery occlusion. In the RIC group, the rats underwent the same operation as the I/R group, in addition to three cycles of 5 min I/R (45 min before ischemia and 15 min after ischemia; Figure 1). Each cycle comprised 5 min of ligation of the right femoral artery (RFA) and 5 min of reflow. In the I/R+Fas group, 10 mg/kg fasudil (a Rho-kinase inhibitor) was injected intravenously in I/R-treated rats 5 min before reperfusion. In the RIC+Wort group, 15 *μ*g/kg wortmannin (a PI3K inhibitor) was injected 5 min before reperfusion in RIC-treated rats. In the RIC+Fas+Wort group, RIC-treated rats were injected with fasudil (10 mg/kg) and wortmannin (15 *μ*g/kg) consecutively 5 min before reperfusion (Figure 1).

### 2.4. Measurement of Myocardial Enzymes

After 180 min of reperfusion, a disposable heparin sodium tube was used to collect arterial blood sample from the common carotid artery. Blood sample was then centrifuged at 1509 × *g* for 15 min to obtain plasma. The activity of LDH and CK was measured by assay kits. The content of cTnI was measured using ELISA kits following the manufacturer's instructions.

### 2.5. Histopathology of Myocardial Infarction Tissue

At the end of reperfusion, the heart was immediately removed and washed in phosphate-buffered saline. The heart was then fixed in the Langendorff perfusion system, and the coronary artery was ligated *in situ* after washing. One portion of the myocardial infarction tissue was immersed in 4% paraformaldehyde, fixed with paraffin, and then cut into 5 *μ*m thick sections. The sections were then deparaffinized, stained with hematoxylin and eosin (H&E), and structural abnormalities were analyzed under an Olympus BX40microscope (Olympus Optical, Tokyo, Japan). For quantitative pathological statistical analysis of cardiac damage, sections (*n* = 4 for each group) were scored by 2 independent observers blinded to the experimental protocol. The damage scores were classified as follows [30, 31]: 0, no abnormality; 1, interstitial edema; 2, cardiomyocyte edema, most of the myocardial fibers arranged regularly; 3, cardiomyocytes arranged disorderly, with occasional red blood cell extravasation and inflammatory cell infiltration; and 4, myocardial fibers disorganized and ruptured, with inflammatory cell infiltration.

### 2.6. Ultrastructural Assessment of Myocardial Infarction Tissue

For ultrastructural measurement, the myocardial tissue was dissected into 1 mm^3^ pieces, which were then fixed in 2.5% glutaraldehyde and soaked in a solution of 1% osmium tetroxide for 1 h. The fixed tissue was embedded in epoxy resin and sectioned into 70 nm ultrathin sections. The sections were stained with uranyl acetate and lead citrate and immediately imaged by a JEM-1230 transmission electron microscope (JEOL Ltd., Tokyo, Japan).

### 2.7. Western Blotting Analysis

Total protein of myocardial tissue was extracted, and the protein content was quantified using the bicinchoninic acid (BCA) protein determination kit (Beyotime Institute of Biotechnology, Shanghai, China). Equal amounts of protein samples were subjected to 12% and 8% sodium dodecyl sulfate-polyacrylamide gel electrophoresis. Then, the gels were transferred onto polyvinylidene difluoride membranes. Next, the membranes were immersed with 5% nonfat milk powder in TBST (Tris-buffered saline with 1% Tween-20) for 2 h. Membranes were incubated overnight at 4°C with mouse anti-LC3-II (1 : 500), p-MLC (1 : 2000), rabbit anti-MLC (1 : 500), p-MYPT1 (1 : 2000), MYPT1 (1 : 500), p-AKT (1 : 8000), AKT (1 : 1000), Beclin 1 (1 : 2000), and GAPDH (1 : 5000). After washing three times with TBST, the membranes were incubated with the corresponding horseradish peroxidase- (HRP-) conjugated goat anti-rabbit secondary antibody (1 : 8000) and goat anti-mouse secondary antibody (1 : 4000) for 1 h at room temperature. After three washes with TBST, the membranes were exposed to Bio-Rad electrophoresis (Bio-Rad Laboratories, Hercules, CA, USA). All band intensities were analyzed by ImageJ software.

### 2.8. Statistical Analysis

Statistical analyses were performed using SPSS22.0 software (IBM Corporation, Armonk, NY, USA). The results were expressed as the mean ± standard deviation (SD). Data were analyzed by one-way analysis of variance and the *q* test. *P* < 0.05 was regarded as statistically significant.

## 3. Results

### 3.1. Changes in MAP and HRat Different Time Points after I/R

**Figure 2:**
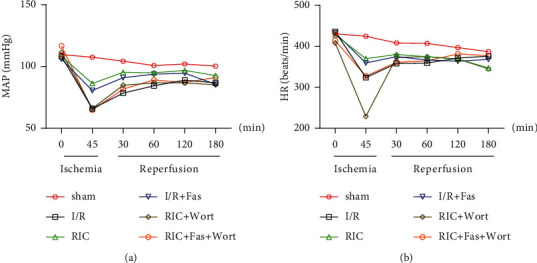
Changes in MAP (a) and HR (b) in the different groups. HR: heart rate; MAP: mean arterial pressure; sham: LAD with no other intervention; I/R: ischemia/reperfusion; RIC: remote ischemic conditioning; Fas: fasudil; Wort: wortmannin. Data are presented as the mean ± SD (*n* = 10).

Consistent with previous studies, myocardial I/R resulted in a significant decrease in MAP and HR. As shown in Figure 2, the decrease was most obvious at 45 min of ischemia and 30 min and 60 min of reperfusion. Interestingly, MAP and HR were markedly improved in the RIC and I/R + Fas groups. Specifically, MAP was significantly higher at 45 min of ischemia and at 30 and 60 min of reperfusion in the RIC and I/R+Fas groups compared with the I/R group, whereas HR was only higher at 45 min of ischemia. Notably, treatment with the PI3K/AKT inhibitor wortmannin markedly diminished the RIC effect, as evidenced by the significantly lower MAP at 45 min of ischemia and at 30 min of reperfusion in the RIC+Wort and RIC+Fas+Wort groups compared with the RIC group (Figure 2(a)). However, HR was only significantly lower at 45 min of ischemia in the RIC+Wort group compared with the RIC group (Figure 2(b)).

### 3.2. Alterations in LDH, CK, and cTnI Levels in the Plasma

**Figure 3:**
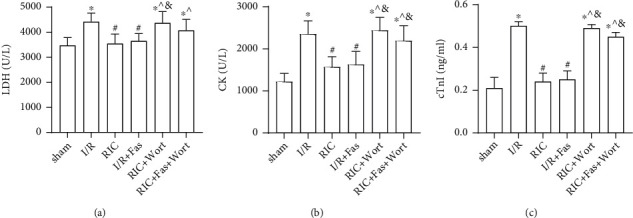
Changes in plasma LDH (a), CK (b), and cTnI (c) in the different groups. LDH: lactate dehydrogenase; CK: creatine kinase; cTnI: cardiac troponin; Data are presented as the mean ± SD (*n* = 10). ^∗^*P* < 0.05 vs. sham group; ^#^*P* < 0.05 vs. I/R; ^*P* < 0.05 vs. RIC; ^&^*P* < 0.05 vs. I/R+Fas group.

As essential indicators of myocardial damage, plasma LDH, CK, and cTnI levels were monitored after I/R. After 45 min of ischemia and 180 min of reperfusion, the levels of LDH and CK and the content of cTnI were significantly higher in the I/R group compared with the sham group (Figure 3), indicating the successful establishment of the I/R injury model. In contrast, the levels of LDH and CK, and the content of cTnI were significantly lower in the RIC and I/R+Fas groups compared with the I/R group. There were no significant differences in these enzymes between the RIC and I/R+Fas groups, which indicated that both RIC and the Rho-kinase inhibitor improved myocardial injury. However, the levels of these enzymes were higher in the RIC+Wort and RIC+Fas+Wort groups compared with the RIC and I/R+Fas groups. Moreover, we found that the level of CK and the content of cTnI were elevated in the RIC+Fas+Wort group compared with the I/R+Fas group, indicating that the cardioprotection of RIC or fasudil was suppressed by wortmannin (Figures 3(a)–3(c)).

### 3.3. Histological Changes in the Myocardial Tissue

**Figure 4:**
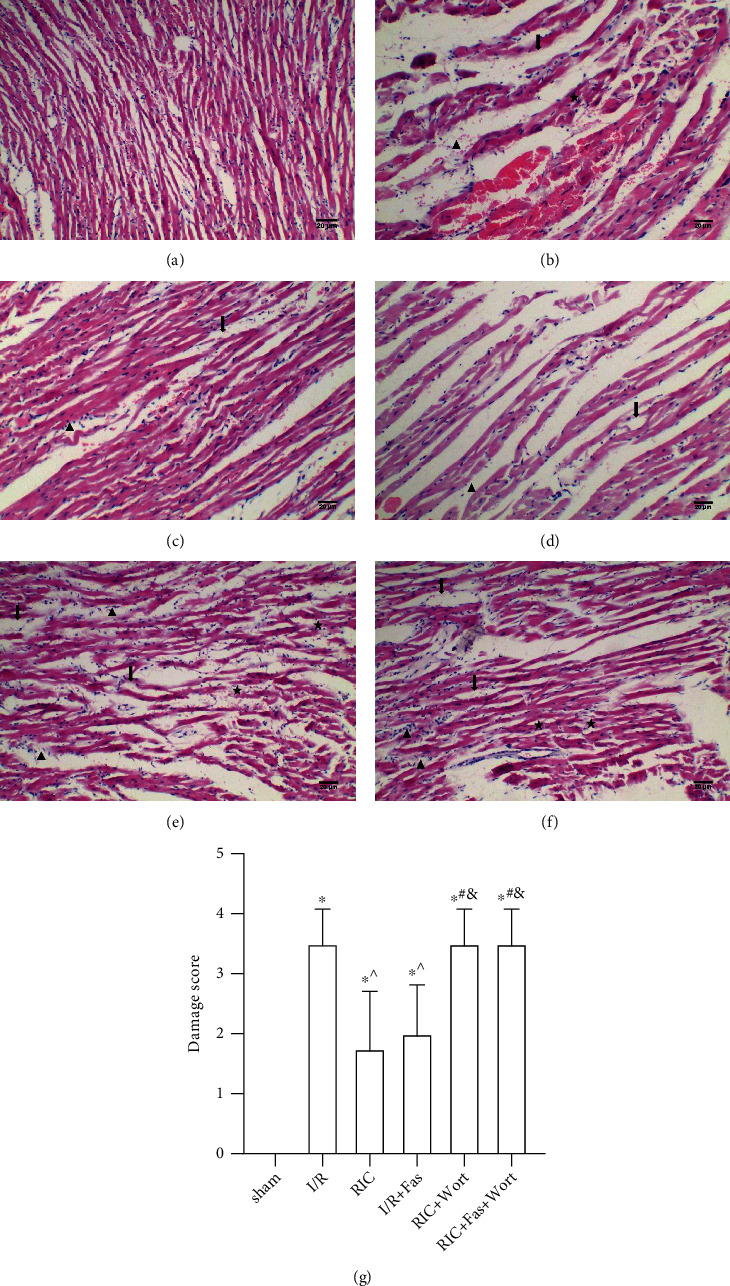
The histological changes in the myocardial tissue in the different groups (×100). Scale bar, 20 *μ*m. (★) myocardial fiber disorganization; (↓) hemorrhage, stromal edema; (▲) inflammatory cell infiltration. (a) Sham group; (b) I/R group; (c) RIC group; (d) I/R+Fas group; (e) RIC+Wort group; (f) RIC+Fas+Wort group; (g) damage score in different groups (*n* = 4). ^∗^*P* < 0.05 vs. sham group; ^^^*P* < 0.05 vs. I/R group; ^#^*P* < 0.05 vs. RIC group; ^&^*P* < 0.05 vs. I/R+Fas group.

H&E staining of sham group myocardial tissue samples showed intact myocardial fibers and a clear structure, without inflammatory infiltration (Figure 4(a)). In the I/R group, myocardial fiber disarrangement, inflammatory cell infiltration, and enlarged intercellular space were observed, which indicated that I/R had destroyed the myocardial tissue (Figure 4(b)). In the RIC and I/R+Fas groups, these changes were alleviated compared with the I/R group with a notably lower damage score. The myocardial cells were arranged relatively regularly; hemorrhage, stromal edema, and inflammatory infiltration were alleviated (Figures 4(c) and 4 (d)). However, wortmannin treatment aggravated these histological parameters in the RIC+Wort and RIC+Fas+Wort groups (Figures 4(e) and 4(f)).

### 3.4. Ultrastructural Changes in Myocardial Fibers and Mitochondria

**Figure 5:**
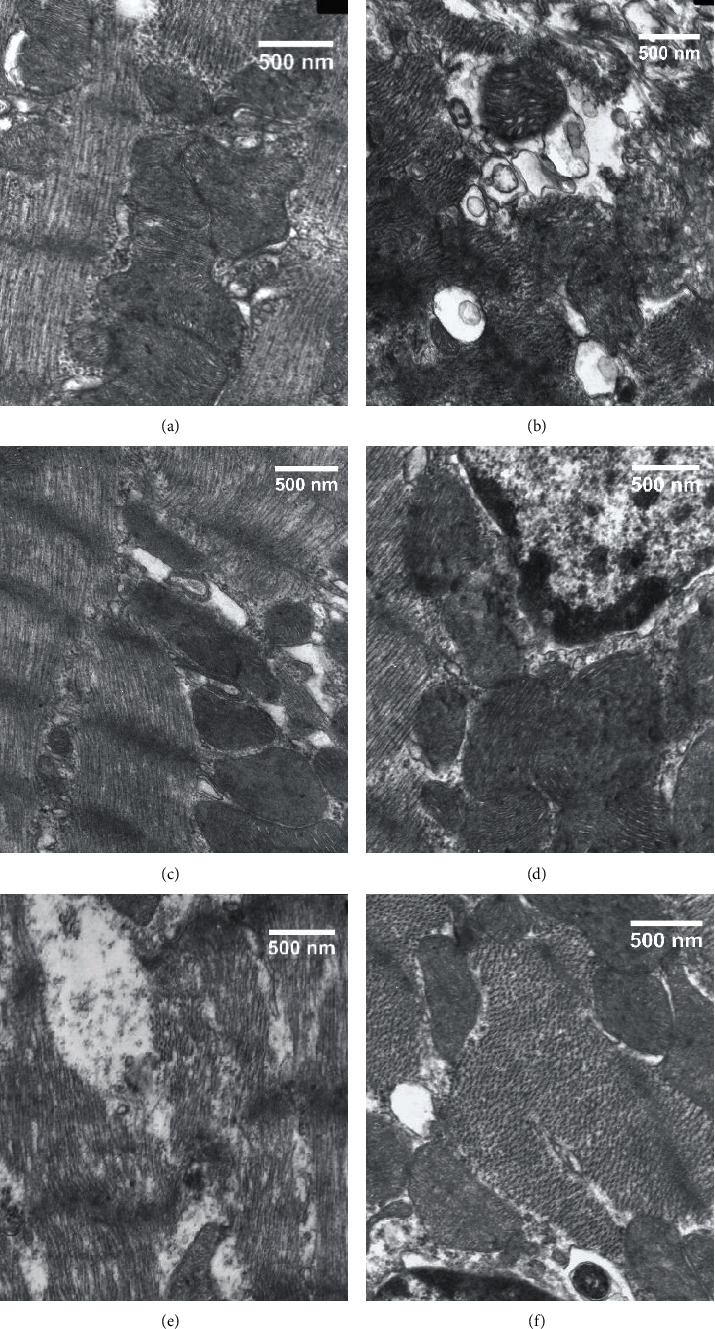
Ultrastructural changes in myocardial fibers and mitochondria in the different groups (×15,000). Scale bar, 500 nm. (a) Sham group; (b) I/R group; (c) RIC group; (d) I/R+Fas group; (e) RIC+Wort group; (f) RIC+Fas+Wort group.

An ultrastructural examination of the sham group showed that the myocardial fibers and sarcomeres were neatly arranged and the mitochondrial cristae were clear and intact (Figure 5(a)). In the I/R group, the myocardial ultrastructure was destroyed, the myocardial fibers were irregularly arranged, and the mitochondria were swollen (Figure 5(b)). As shown in Figures 5(c) and 5(d), compared with the I/R group, the muscle sarcomeres were arranged in a relatively neat way with clear Z lines and the mitochondria were intact in the RIC and I/R+Fas groups. When treated with wortmannin, the myofilaments were partly ruptured and the sarcomeres were disordered in the RIC+Wort and RIC+Fas+Wort groups (Figures 5(e) and 5(f)).

### 3.5. Changes in p-MLC, p-MYPT1, Beclin 1, LC3-II, and p-AKT Protein Levels in the Myocardial Tissue

**Figure 6:**
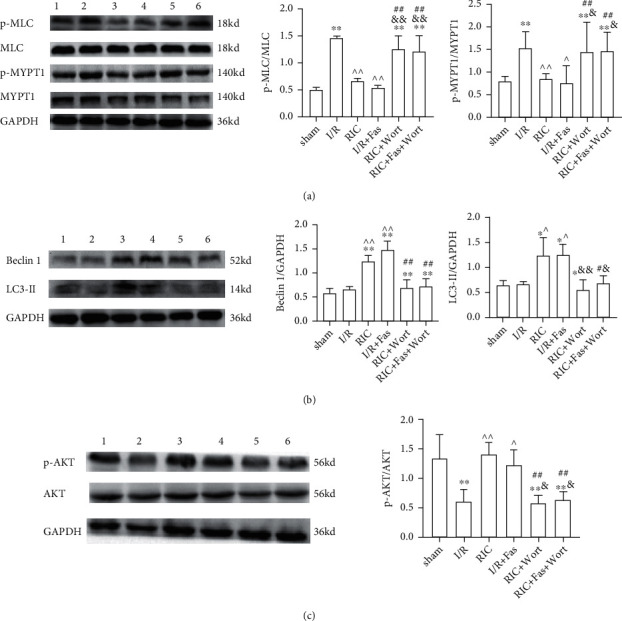
The protein expression and the ratio of p-MLC and p-MYPT1 (a), Beclin 1 and LC3-II (b), and p-AKT (c) in the myocardium in the different groups. Lane 1: sham group; lane 2, I/R group; lane 3, RIC group; lane 4, I/R+Fas group; lane 5, RIC+Wort group; lane 6, RIC+Fas+Wort group. Data are presented as the mean ± SD (*n* = 4). ^∗^*P* < 0.05 vs. sham group; ^∗∗^*P* < 0.01 vs. sham group; ^#^*P* < 0.05 vs. RIC group; ^##^*P* < 0.01 vs. RIC group; ^^^*P* < 0.05 vs. I/R group; ^^^^*P* < 0.01 vs. I/R group; ^&^*P* < 0.05 vs. I/R+Fas group; ^&&^*P* < 0.01 vs. I/R+Fas group.

To clarify the RIC underlying mechanism, we examined the role of Rho-kinase in the pathological development of myocardial ischemia. The protein expression levels of p-MLC and p-MYPT1 in ischemic heart tissue were determined (Figure 6(a)). The results showed that compared with the sham group, the expression levels of p-MLC and p-MYPT1 were markedly higher in the I/R, RIC+Wort, and RIC+Fas+Wort groups. However, compared with the I/R group, the expression levels of p-MLC and p-MYPT1 were significantly lower in the RIC and I/R+Fas groups. These results showed that both RIC and Rho-kinase inhibitor reduced the expression of Rho-kinase, while wortmannin abrogated this effect. Furthermore, we examined the effects of autophagy on the myocardium. Our results showed that Beclin 1 and LC3-II protein expression were enhanced in the RIC and I/R+Fas groups compared with the I/R group, which indicated that RIC and fasudil induced autophagy to protect cardiomyocytes (Figure 6(b)). Consistently, PI3K inhibition by wortmannin decreased this protective effect of RIC. To verify the role of the PI3K/AKT signaling pathway, we also examined the expression levels of p-AKT in each group. As expected, the expression levels of p-AKT in the I/R, RIC+Wort, and RIC+Fas+Wort groups were lower than in the sham group, but in the RIC and I/R+Fas groups, it was similar to the sham group level (Figure 6(c)). In summary, our results demonstrated that RIC treatment enhanced autophagy through the PI3K/AKT signaling pathway.

## 4. Discussion

MIRI induces myocardial inflammation and oxidative stress, aggravates structural dysfunction, and ultimately exacerbates cell death [32, 33]. RIC is a novel method for alleviating this damage. This study investigated the effect and mechanism of Rho-kinase on MIRI in rats treated with RIC. We found that RIC protected the myocardium by inhibiting Rho-kinase activity and enhancing intracellular autophagy. After inhibiting the activity of PI3K, the activity of Rho-kinase in the RIC group was enhanced, autophagy was weakened, and myocardial injury was aggravated. These findings indicated that RIC prevented I/R injury by inhibiting Rho-kinase and inducing autophagy in cardiomyocytes through the PI3K/AKT pathway.

RIC is a novel conditioning approach for treating MIRI that involves applying transient I/R episodes to a tissue that is remote from the ischemic myocardium. Although RIC is used in the clinical treatment of ischemic diseases, the related mechanisms need to be further investigated [23]. During I/R, the myocardial membrane integrity is lost, and myocardial enzymes such as LDH, CK, and cTnI are released into the plasma. Therefore, plasma LDH, CK, and cTnI are usually determined as indicators of myocardial tissue damage. In the current study, the LDH, CK, and cTnI levels were higher, while the MAP and HR levels were lower in the I/R group compared with the sham group. Additionally, the myocardial structure of the I/R group was disorganized, with inflammatory cell infiltration and mitochondrial swelling. However, RIC treatment reversed these effects, which highlighted the myocardial protection of RIC as shown in previous studies [2, 3, 15]. Interestingly, there were no differences in myocardial enzymes and structure function between RIC application and fasudil treatment of I/R rats. These findings suggested that RIC and Rho-kinase inhibitor mitigated myocardial damage to reduce I/R injury.

The Rho/Rho-kinase signaling pathway participates in the pathological development of myocardial diseases [34]. MLC and MYPT1 are the most characteristic substrates of Rho-kinase. Activated Rho-kinase increases the phosphorylation of MLC and MYPT1, leading to excessive contraction of vascular smooth muscles. This process eventually aggravates myocardial damage. Therefore, the levels of p-MLC and p-MYPT1 are usually used to reflect Rho-kinase activity [34, 35]. Our previous study showed that p-MLC and p-MYPT1 protein expression levels were significantly reduced in the I/R model after RIPostC treatment [3]. The present study showed that in the RIC group, myocardial enzymes and inflammatory infiltration were reduced, the myocardial structure and function were stable, and the p-MLC and p-MYPT1 protein expression levels were decreased compared with the I/R group; similar findings were observed in the I/R+Fas group. These findings indicated that the cardioprotective effect of RIC was mediated by inhibiting Rho-kinase activity.

Autophagy acts as a cellular defense mechanism against bacterial toxins. Autophagy participates in physiological processes in ischemic cardiomyopathy, such as cell activity, inflammation, and oxidative stress [19]. Some studies have shown that autophagy elicited by hypoxia or ischemia may provide protection against cardiac injury [36, 37] and that enhanced autophagy protected against I/R injury in cardiac myocytes [30]. In another study, a reduction in autophagy by RNA interference of Beclin 1, which is involved in mediating autophagy, enhanced cardiac cell survival [38]. To address the controversial role of autophagy in the myocardium, we examined the function of autophagy in RIC. We found that LC3-II and Beclin 1 protein expression was increased after RIC treatment and that myocardial injury was reduced, which indicated that RIC induced autophagy to protect cardiomyocytes. Similarly, fasudil treatment increased LC3-II and Beclin 1 protein expression in I/R rats and suppressed p-MLC and p-MYPT1 protein expression, suggesting that inhibition of Rho-kinase also enhanced autophagy to protect the myocardium. Additionally, after inhibiting PI3K with wortmannin in the RIC group, the expression of LC3-II and Beclin 1was reduced, indicating that the protective effect of RIC was related to the PI3K pathway and that this pathway was upstream of autophagy.

The PI3K/AKT pathway is a crucial regulator of the reperfusion injury salvage kinase (RISK) pathway. The PI3K/Akt signaling pathway has a vital role in cardioprotection by inducing proliferation and survival and reducing inflammation and oxidative stress [22, 33]. In our study, we found that p-AKT expression was increased after RIC treatment, which suggested that RIC induced the PI3K/AKT pathway. To examine the mechanism, we added wortmannin to the RIC model. The results showed that p-MLC and p-MYPT1 protein expression was enhanced, while p-AKT protein expression was decreased, and that the cardioprotection of RIC was attenuated, suggesting that inhibition of the PI3K/AKT pathway decreased the cardioprotective effect of RIC by regulating Rho-kinase activity. To further determine the relationship between Rho-kinase and the PI3K/AKT pathway in RIC treatment, we combined fasudil and wortmannin in RIC-treated rats. We found that when the PI3K/AKT pathway was inhibited, the inhibitory effect of fasudil on Rho-kinase was also abolished in the RIC rats. Moreover, the expression of p-MLC and p-MYPT1 was enhanced, and the myocardial injury was elevated with the increase in myocardial enzymes, as well as the dysfunction of myocardial structure and function, and the decrease in autophagy-related proteins' expression. These data provided evidence that when wortmannin inhibited the PI3K/AKT pathway in the RIC group, inhibiting Rho-kinase by fasudil did not induce the cardioprotective effect. This suggested that RIC prevented I/R injury by inhibiting Rho-kinase and that the PI3K/AKT pathway participated in the regulation of Rho-kinase. However, further investigation is needed to analyze this relationship.

Nevertheless, there are some limitations in the present study. The signaling pathways and regulation mechanisms involved in the cardioprotection of RIC are complicated. In this study, we mainly focused on the relationship of Rho-kinase and the PI3K/AKT signaling pathway in the RIC-induced protection, therefore, and we only examined the expression of autophagy-related proteins (e.g., LC3-II and Beclin 1). Further studies are required to determine how autophagy is regulated in RIC. Furthermore, our study demonstrated that an interaction may exist between Rho-kinase and the PI3K/AKT pathway during RIC protection, however investigating whether Rho-kinase and the PI3K/AKT pathway have a dual-direction regulation in RIC protection is required to deliver further interesting insights in the future.

## 5. Conclusions

**Figure 7:**
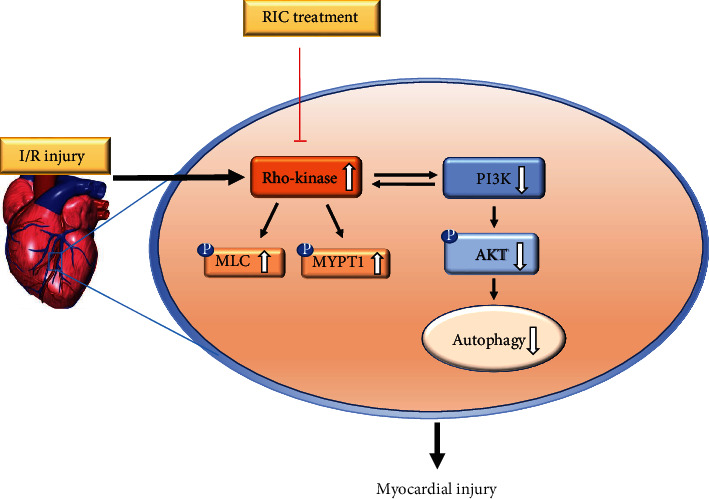
Schematic diagram summarizing the possible mechanisms of Rho-kinase and the PI3K/AKT pathway in RIC.RIC is a novel therapy that protects against myocardial I/R injury by inhibiting Rho-kinase activity and activating the PI3K/AKT pathway to enhance autophagy. Black solid arrows indicate positive effects, red lines with vertical bars indicate negative effects, and black hollow arrows indicate up or down regulation.

This study showed that Rho-kinase and the PI3K/AKT pathway could contribute to RIC protection against I/R injury. As illustrated in Figure 7, our findings provided support for future research. These results indicated that the cardioprotective role of RIC was mediated by inhibition of Rho-kinase in association with upregulation of autophagy in the myocardium.

## Data Availability

The datasets used and/or analyzed during the current study are available from the corresponding author upon reasonable request.
